# Heart Failure in Older Adults: Medical Management and Advanced Therapies

**DOI:** 10.3390/geriatrics7020036

**Published:** 2022-03-23

**Authors:** Ellen Liu, Brent C. Lampert

**Affiliations:** Division of Cardiovascular Medicine, The Ohio State University Wexner Medical Center, Columbus, OH 43210, USA; ellen.liu@osumc.edu

**Keywords:** heart failure, elderly, polypharmacy, heart transplant, ventricular assist device

## Abstract

As the population ages and the prevalence of heart failure increases, cardiologists and geriatricians can expect to see more elderly patients with heart failure in their everyday practice. With the advancement of medical care and technology, the options for heart failure management have expanded, though current guidelines are based on studies of younger populations, and the evidence in older populations is not as robust. Pharmacologic therapy remains the cornerstone of heart failure management and has improved long-term mortality. Prevention of sudden cardiac death with implantable devices is being more readily utilized in older patients. Advanced therapies have provided more options for end-stage heart failure, though its use is still limited in older patients. In this review, we discuss the current guidelines for medical management of heart failure in older adults, as well as the expanding literature on advanced therapies, such as heart transplantation in older patients with end-stage heart failure. We also discuss the importance of a multidisciplinary care approach including consideration of non-medical co-morbidities such as frailty and cognitive decline.

## 1. Introduction

With the advancement of medicine, technology, and preventative care, the average life expectancy at birth has increased and is expected to continue rising. Worldwide, there has been an increase in the population 65 years and older [1,2,3,4] as well as the very elderly population greater than 85 years old [5]. By 2050, it is estimated that 17 percent of the world population and 21 percent of the United States (US) population will be above the age of 65 years [4,6]. Within the US, average total life expectancy is expected to reach 85.6 years by 2060 [1]. With the aging population, cardiologists and geriatricians can expect to see an increase in the number of older patients with multimorbidity and cardiovascular disease.

The prevalence of heart failure increases significantly with age and is estimated to affect more than 6 million adults in the US [3]. Prevalence almost doubles from approximately 6 percent of the population between 60 and 79 years to 11 percent of those greater than 80 years old [3]. The higher prevalence in the older population is likely attributed to age-related heart failure risk factors such as hypertension, coronary artery disease, diabetes, and structural and functional changes. Age plays a role in clinical characteristics and prognostic factors and outcomes in older patients. Older patients hospitalized with heart failure are more often female and have a higher prevalence of preserved ejection fraction and more significant valvular disease, though lower rates of traditional risk factors such as ischemic heart disease and diabetes [7]. Additionally, heart failure in older adults is associated with cognitive decline, frailty, and malnutrition and can significantly decrease quality of life [8]. The advancement of medicine and technology has resulted in a wider-range of treatment options for patients with heart failure. However, even though half of all patients with heart failure are over 75 years old, most clinical trials assess much younger cohorts. Applying conventional guidelines formulated using trial data derived from younger populations may call for special considerations for older patients, but often they do not provide concrete evidence-based recommendations. It is important to consider how age, comorbidities, and geriatric syndromes impact the management options, including medical and advanced therapies, for our older patients. This review was developed through an extensive PubMed search and review of pivotal trials of guideline-directed medical and device therapy for heart failure, with a specific focus on those addressing the geriatric population, polypharmacy, cognitive decline, and frailty.

## 2. Medical Management

### 2.1. Pharmacologic Therapy

Long-term heart failure mortality has improved with guideline-directed pharmacologic therapy and treatment of risk factors, though absolute mortality remains approximately 50% within five years of diagnosis [3]. Current heart failure guidelines [9,10] provide a number of Class I recommendations for medications in the management of heart failure with reduced ejection fraction, including renin–angiotensin receptor blockers or angiotensin receptor neprilysin inhibitors, beta blockers, aldosterone receptor antagonists, and loop diuretics for patients with evidence of fluid retention (Central Figure). Additionally, the sodium–glucose cotransporter-2 inhibitors dapagliflozin and empagliflozin were recently FDA approved for the management of heart failure with reduced ejection fraction [11,12]. These medications appear to also be beneficial in heart failure with preserved EF [13,14], which is particularly important given its increased prevalence in the elderly population. 
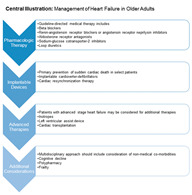


Patients greater than 75 years old are under-represented in pivotal trials supporting the use of guideline-directed medical therapies. These trials also excluded those who did not meet criteria for creatinine clearance, heart rate, and systolic blood pressure. For example, the US Carvedilol Heart Failure study excluded patients with a systolic blood pressure greater than 160 mmHg and heart rate less than 68 beats per minute, and the average age of participants was 58 years old [15]. The SOLVD study evaluating the benefit of enalapril excluded patients greater than 80 years old and those who had an elevated creatinine or “any other disease that might substantially shorten survival or impede participation in a long-term trial [16]”. The average age of participants was 61 years old. While these trials have demonstrated mortality benefit with use of the studied medications, it serves as a reminder that trial populations often do not reflect real world patient populations. Pharmacologic therapy needs to be carefully selected in older adults for many reasons. With aging, there are changes in drug metabolism and patient body composition that can impact volume of distribution and plasma concentrations of medications. Older patients are also more likely to have concomitant renal or liver disease that raise the risk of medication-related adverse events. The presence of sick sinus syndrome and orthostatic hypotension may also impact the appropriateness of pharmacologic therapy in older patients. In the absence of clear guidance on when to discontinue or how to prioritize pharmacologic therapy in older patients, many clinicians continue to prescribe these medications despite a risk–benefit ratio that is less than clear. Target doses for older populations are not well established, and one meta-analysis suggested no benefit of angiotensin converting enzyme inhibitors after age 75 [17]. The potential of adverse effects should be routinely assessed when balancing the risks and benefits of pharmacologic therapies, particularly when life expectancy is shorter, decreasing the time horizon for potential benefit from pharmacologic therapy. This should include ongoing assessment of an older patient’s quality of life and priorities for medical care—living longer, maintaining current health, or comfort.

### 2.2. Defibrillators and Cardiac Resynchronization Therapy

In addition to pharmacologic therapy, primary prevention of sudden cardiac death is a routine part of guideline-directed therapy in select patients with cardiomyopathy and heart failure. Implantable cardioverter-defibrillator (ICD) therapy is recommended for patients with a left ventricular ejection fraction of 35% or less and New York Heart Association (NYHA) class II or III symptoms. Cardiac resynchronization therapy (CRT) is recommended for patients with a left ventricular ejection fraction of 35% or less and left bundle-branch block with a QRS duration of greater than 150 milliseconds, in addition to NYHA class II–IV symptoms [10]. Notably, the guidelines currently do not provide an age cutoff for those who should receive ICD and CRT implantations.

Although the mortality benefits of these devices have been shown in landmark trials [18,19,20,21], older adults were not well represented in the treatment groups, with the average age being between 58 and 64 years across these studies. Nonetheless, more than 40% of new ICDs and CRTs are implanted in patients >70 years old and more than 10% in patients >80 years old [22,23]. ICD therapy can reduce mortality, but it does not have any effect on symptoms or quality of life. In contrast, older patients have been shown to benefit symptomatically and echocardiographically from cardiac resynchronization similar to their younger counterparts [24,25]. From a mortality standpoint, the etiology of cardiomyopathy likely drives the benefit older patients receive from the addition of a defibrillator to resynchronization therapy. A study by Wang et al. found no difference in survival benefit in patients ≥75 years old with non-ischemic cardiomyopathy [26]. Gras et al. evaluated patients with ischemic and non-ischemic cardiomyopathy and found improved all-cause death in patients >75 years old with ischemic cardiomyopathy, whereas no benefit was seen in those with non-ischemic cardiomyopathy [27].

It is important to note that recommendations for device therapy are for patients with an expected meaningful survival of greater than one year, though decision making can be challenging given concurrent comorbidities, geriatric conditions, and how “meaningful survival” is defined. Additionally, procedural complications and routine maintenance and monitoring of implanted devices must be considered. As such, it is important to assess patients on an individualized basis when deciding who would be an appropriate candidate to refer for defibrillator or cardiac resynchronization therapies. Decision aids for ICDs are available and can be helpful in facilitating these conversations [28]. It would be reasonable to pursue placement of an ICD in an older patient with a life expectancy greater than one year and meaningful quality of life. For older patients with an ICD already in place, it can be important to discuss whether to deactivate shock therapy as their medical condition declines to avoid painful shocks at the end of life. Conversation around the risks and benefits of generator change is also important when batteries reach end of life.

## 3. Advanced Therapies

Despite evidence-based medical and device-based therapies, about 10 percent of heart failure patients progress to “advanced” or end-stage heart failure. Advanced heart failure is characterized by progressive symptoms including exercise intolerance, weight loss, refractory volume overload, and hypotension [29]. When heart failure reaches the advanced stage, numerous treatment options remain, including intravenous inotropes, left ventricular assist devices, and cardiac transplantation. However, these therapies all carry trade-offs in the form of serious adverse events or loss of independence that can impact quality of life. Thorough assessment of patient comorbidities and discussions about patient and caregiver preferences are critical for assessing the appropriateness of these therapies in the elderly.

### 3.1. Inotropes

Positive inotropic drugs such as dobutamine and milrinone improve hemodynamics in decompensated heart failure with low cardiac output and evidence of poor end-organ perfusion. They are commonly used short-term in patients with cardiogenic shock to maintain systemic perfusion while the acute precipitating event is treated or to bridge patients to a more durable advanced therapy. Due to concerns regarding increased mortality associated with their use, long-term inotropes are not routinely recommended. However, for select patients who cannot be weaned from inotropes or who receive significant symptomatic benefit from their use, long-term inotropes can be considered. Accordingly, current heart failure guidelines provide a Class IIb recommendation for long-term ambulatory use of inotropes as a palliative option for patients with end-stage heart failure who are not candidates for advanced therapies [10]. While continuous inotropes have be shown to be associated with higher mortality and adverse events [30,31,32], their use for improving symptoms and reducing hospitalizations is appropriate in the palliative setting [33,34,35,36]. Although there are limited data focused on the effects of inotropes in the elderly, given the limited options older patients with end-stage heart failure face, inotropes can be a therapeutic option for carefully selected patients. When utilizing inotropes, palliative care assistance should be considered to help manage symptoms and develop an end of life care plan.

### 3.2. Ventricular Assist Devices

Improvement in mechanical circulatory support technology has led to an increase in left ventricular assist device (LVAD) implantations, decreased in-hospital mortality, and improved survival [37,38]. The average patient age at LVAD implant remains approximately 57 years old [38], but as the population of patients with end-stage heart failure ages, the number of older patients undergoing LVAD implants for destination therapy is increasing. Analysis of data from the National Inpatient Sample found that the number of LVAD implants in patients greater than 65 years old has increased from 20% in 2007 to 33% in 2014 [39] and that adults greater than 75 years old receiving implants increased from 3.5% in 2003 to 10.5% in 2014 [40]. Data from the Interagency Registry for Mechanically Assisted Circulatory Support (INTERMACS) reveal that the proportion of patients greater than 75 years receiving an LVAD has remained approximately 5% in recent years [41].

Existing data regarding outcomes in older adults with LVADs remain limited and contradictory. Analyses of INTERMACS database and National Inpatient Sample of older adults concluded that age remains a predictor of poor outcomes after LVAD implantation [41,42]. Patients greater than 75 years have lower survival rates and are less likely to be discharged home compared with patients younger than 55 years [41]. In contrast, an analysis of the Mechanical Circulatory Support Research Network registry revealed that age greater than 70 years was not a strong predictor of mortality, with a one-year survival similar to all destination therapy patients [43]. Another study of patients greater than 70 years old who received the HeartMate II LVAD showed good functional recovery, survival, and quality of life at two years, concluding that age alone should not be a contraindication for LVAD therapy [44].

Potential complications such as bleeding, which older patients are more likely to suffer from [41,43], infection, stroke, and pump thrombosis are additional factors to consider when deciding whether your older patient would be a candidate for LVAD. However, significant recent advances in LVAD technology have improved outcomes and hemocompatibility-related adverse events [45]. While survival with newer-generation LVADs remains lower in the elderly when compared with younger populations, elderly patients have shown similar improvements in quality of life and functional capacity, while having a lower rate of late major complications [46]. Despite these encouraging findings, use of LVADs in the elderly has not increased in the most recent era [46]. Proactive discussions, with the assistance of palliative care, about the risks and benefits of LVAD therapy in the elderly are critical to allow for timely implantation before elderly patients become too frail or have other end-organ dysfunction that will limit successful outcomes or eliminate potential LVAD candidacy.

### 3.3. Cardiac Transplantation

**Figure 1:**
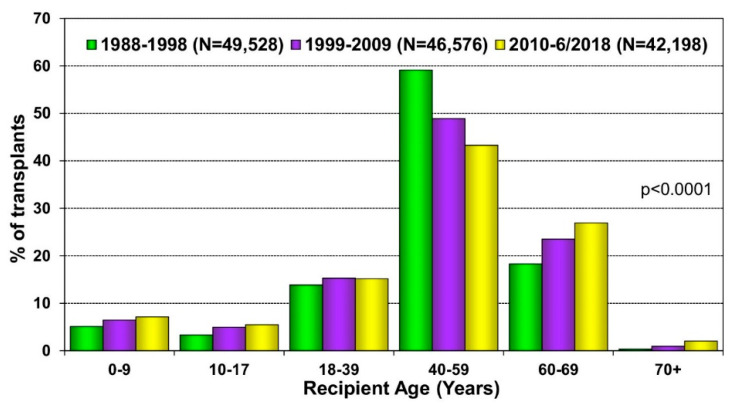
Recipient age distribution (adult and pediatric) by era. Reprinted with permission from Khush KK, Wida CS, Chambers DC, et al. The International Thoracic Organ Transplant Registry of the International Society for Heart and Lung Transplantation: Thirty-sixth adult heart transplantation report—2019; focus theme: Donor and recipient size match. J Heart Lung Transplant; 2019; 38: 1056–1066.

Cardiac transplantation remains the best therapeutic option for patients with end-stage heart failure. In the early era of heart transplantation, older patients were not considered transplant candidates, and further screening was recommended for patients over the age of 50 years old [47]. Due to improved outcomes in older patients and advances in post-transplant care, the 2006 International Society for Heart Lung Transplantation listing criteria for heart transplantation provided a Class IIb recommendation for carefully selected patients greater than 70 years old to be considered for transplantation [48], a recommendation that has carried onto the most recent 2016 guidelines [49]. The change in recommendations was based on data demonstrating comparable survival in older patients compared with younger patients. Weiss et al. reviewed the United Network for Organ Sharing database and found the five-year survival of patients greater than 60 years old to be nearly 70%—an acceptable rate compared with 75% in younger patients [50]. Goldstein et al. found that although patients in their 70s had lower survival compared with patients in their 60s, they still benefited significantly from cardiac transplant, with a median survival of 8.5 years compared with 9.8 years in the younger patients [51]. Other studies have found that the outcomes of transplant recipients in their 70s were similar to recipients in their 60s without significant difference in morbidity or mortality [52,53]. Additionally, older patients were less likely to experience rejection than their younger cohort [51,52]. These studies have suggested that age itself should not be an exclusion criterion to be considered for transplantation and that the proportion of heart transplants in older patients has been steadily increasing (Figure 1). However, careful consideration of a patient’s other end-organ function, frailty, and ability to recover from major surgery are a critical part of the evaluation for heart transplantation in the older population.

## 4. Additional Considerations

When caring for older patients with heart failure, a multifactorial approach should be taken that includes assessment and management of associated geriatric syndromes such as cognitive impairment, frailty, and malnutrition, which can play a role in hospitalization, quality of life, morbidity, and mortality.

### 4.1. Polypharmacy

Polypharmacy, defined as five or more medications, is nearly universal in heart failure patients due to multimorbidity and guideline-based medication recommendations. Heart failure patients take an average of 10 medications, all of which carry risk for drug interactions and adverse reactions [54]. There is an increasing prevalence of polypharmacy in older adults, estimated to have grown from 24% in 2000 to 39% in 2012 amongst adults 65 years and older [55]. Though many trials have excluded older, multimorbid adults, guidelines do not provide concrete recommendations for discontinuation in older adults, and thus, medications are often continued indefinitely. Increased medication burden can decrease functional capacity and quality of life, as well as increasing the risks of side effects and adverse events. This then can lead to a prescribing cascade [56], which is the prescription of additional medications used to treat the side effects, further increasing the medication burden in older patients.

Recognizing the need to re-examine the benefits and risks of medication use, especially in older adults prone to adverse events, the process known as deprescribing is gaining momentum worldwide. Deprescribing focuses on removing or reducing unnecessary or potentially harmful medication use with the goal of improving outcomes, taking into account an individual’s overall physiologic status, stage of life, and goals of care [56]. Ongoing deprescribing trials focus on populations with higher co-morbidity burden and patients with possible symptoms related to medication use and can examine the safety of deprescribing. For example, the TRED-HF trial was a small, randomized trial of withdrawal of heart failure medications in patients with dilated cardiomyopathy with improved ejection fraction. Patients deemed recovered from dilated cardiomyopathy relapsed following treatment withdrawal, implying that indefinite treatment for cardiomyopathy may be necessary [57]. Although the trial examined a younger population, the implications of the trial could aid in the decision of which medications may or may not be appropriate to discontinue given the high incidence of heart failure in the older population.

For providers and patients interested in pursuing deprescribing, Krishnaswami et al. provide a five-step approach to deprescribing that includes: (1) reviewing and reconciling medications, (2) assessing the risk of adverse drug events, (3) assessing candidacy of individual medications, (4) prioritizing drug discontinuation, and (5) discontinuing medications and implementing monitoring protocols [56]. The decision and process to deprescribe should include patients, their families, and health care personnel such as pharmacists who can help to analyze medication lists and identify potential drug interactions and specific medications to review.

### 4.2. Cognitive Decline

The prevalence of cognitive impairment is approximately 16 to 20 percent of the general population and 40 percent amongst patients with heart failure [58], with an even higher prevalence in patients with more advanced symptoms [59]. Older adults are at risk for not only age-related cognitive changes such as dementia but also cardiovascular etiologies as well, such as cardio-cerebral syndrome in heart failure. The pathophysiology of cognitive impairment in patients with heart failure is multifactorial, and proposed mechanisms include decreased cerebral perfusion and neurohormonal changes [60]. Clinical manifestations include abnormalities in learning, memory, psychomotor speed, executive function, and complex attention [8,61]. Cognitive decline has been associated with worse quality of life, spousal/caregiver distress, readmission risk, and increased mortality risk [62,63]. Reviews of the literature have suggested that while heart failure patients are at increased risk for cognitive decline, this may be modified by treatment such as cardiac transplantation [64,65]. Further investigation to identify the mechanisms of cognitive decline in heart failure and therapeutic interventions is needed. Identification of cognitive impairment through routine screening may help clinicians investigate potential reversible causes, as well as aiding in providing multidisciplinary care.

### 4.3. Frailty

Frailty is a syndrome that is characterized by the exaggerated decline in function and increased physiological vulnerability to stressors [66], commonly seen in older patients with heart failure. The prevalence of frailty ranges from 10% to 60% [66] and is estimated to be approximately 50% in community-dwelling patients with heart failure and 75% amongst hospitalized patients [67]. The biological mechanism of frailty includes up-regulation of inflammatory markers that leads to hormonal dysregulation and a downstream catabolic state and muscle wasting [8]. Frailty has been associated with increased disability, mortality, and hospitalization [68,69]. In a recent prospective study following hospitalized patients greater than 65 years old, advancing age was strongly associated with increased frailty domains, including physical and social frailty, and was associated with higher mortality, heart failure readmissions, and all-cause death [70]. In patients with advanced heart failure, frailty prior to transplantation was found to be associated with increased mortality and hospitalization post-transplant [71].

Frailty assessment is instrumental in helping to guide treatment plans that will maximize patients’ likelihood of a positive outcome and should be implemented as part of routine heart failure management in older adults. The Fried phenotype method (Table 1) is a widely used assessment model, and frailty based on the Fried phenotype is consistently associated with worse clinical outcomes, greater functional impairment, and poor quality of life in older, community-dwelling individuals [72]. An additional importance of recognizing frailty is not just prognostic value. Physical activity is associated with better outcomes, and in those who are frail, increased physical activity can lower the risk of all-cause mortality [73]. The recent REHAB-HF trial found that early, tailored rehabilitation intervention resulted in greater improvement in physical function in older patients hospitalized with decompensated heart failure [74]. Frail patients in particular may benefit from the aforementioned deprescribing strategy described above. This should especially be considered if medical therapy contributes to orthostatic symptoms or fatigue that limits physical rehabilitation or if pill burden limits oral intake that contributes to malnutrition. Unfortunately, no evidence currently exists to guide which medications to prioritize for deprescribing. Individual patient priorities and comorbidities should therefore influence medication changes.

## 5. Conclusions

As the population ages and medical therapy improves, the number of older heart failure patients is expected to increase as well. Although medical therapy has improved outcomes in heart failure, mortality and hospitalization remain high. Older patients were historically underrepresented or excluded from the landmark trials that led to the current heart failure guidelines, though we continue to apply the current recommendations to our older patients.

Heart transplantation remains the best therapeutic option for end-stage heart failure, though age remains a barrier to transplant at many institutions. Alternatively, technological advances have allowed more patients to receive LVAD in end-stage heart failure, but older patients are more prone to complications. Newer LVAD technology may mitigate those complications, but the number of older LVAD patients has not increased in recent years. In our older patients with heart failure, it is also important to take a multifactorial approach to care, while considering geriatric syndromes, polypharmacy, and expected survival when prescribing and offering advanced therapies.

## Figures and Tables

**Table 1:** Fried Frailty Criteria [72].

Frailty Components		Assessment
Weight loss	≥10 pounds unintentional weight loss in the prior year	0 components: non-frail 1–2 components: intermediate risk ≥3 components: frail
Weakness	Grip strength in the lowest 20% adjusted for gender and body mass index	
Exhaustion	Self-reported poor endurance	
Slowness	Slowest 20% of the population based on 15 feet walk time adjusted for gender and height	
Low physical activity	Lowest 20% for each gender	

## Data Availability

Not applicable.
